# Biomechanical comparison of standing posture and during trot between German shepherd and Labrador retriever dogs

**DOI:** 10.1371/journal.pone.0239832

**Published:** 2020-10-02

**Authors:** Alexander Humphries, Aliah F. Shaheen, Constanza B. Gómez Álvarez

**Affiliations:** 1 School of Veterinary Medicine, University of Surrey, Guildford, United Kingdom; 2 Department of Life Sciences, Brunel University London, London, United Kingdom; 3 Department of Veterinary Medicine, University of Cambridge, Cambridge, United Kingdom; University of Lincoln, UNITED KINGDOM

## Abstract

It is widely accepted that canine breeds stand and move differently. The prevalence of various musculoskeletal disorders such as hip and elbow dysplasia is also different between breeds. German shepherd dog (GSD) and Labrador retriever dog (LRD) are two large breeds with different conformations that have high prevalence of these disorders. This study quantifies the movement and standing posture of twelve healthy GSDs and twelve healthy LRDs to identify biomechanical similarities and differences that may be linked to sub-optimal hip and elbow mechanics. A pressure walkway and a motion capture system obtained measures of kinetics, kinematics and conformation during standing and trot. During standing, LRDs carry a greater percentage of the weight on the forelimbs (69%±5% vs. GSDs: 62%±2%, p<0.001) and their body Centre of Pressure (CoP) is located more cranially (p<0.001). GSDs had a greater pelvic tilt (79°±8 vs. 66°±9°, p = 0.004), more flexed stifles (44°±9° vs. LRDs: 34°±10°, p<0.05) and hocks (58°±11° vs. 26°±9°, p<0.01) and more extended hips (-10°±11° vs. 30°±12°, p<0.001). During trot, the GSDs’ CoP had a longer anterior-posterior trajectory (151%±22% vs. LRDs: 93%±25% of the withers height, p<0.001). Stride parameters and loading of limbs were similar when normalised to the size and weight of the dog, respectively. The LRDs had a more extended thoracolumbar angle (p<0.001) and a less flexed lumbosacral angle (p<0.05). The LRDs’ hip remained flexed during trot whereas the GSDs’ hip joint was less flexed during swing (p<0.001) and more extended in late stance and early swing (p<0.001). In conclusion, the LRDs and GSDs differ in the way they stand and move and this would result in different loading pattern of the joints. Further investigation is required to determine the extent to which biomechanical differences are linked to musculoskeletal problems presented clinically.

## Introduction

It is widely accepted that canine breeds stand and move differently depending on their individual breed characteristics [1–3]. A number of pure canine breeds have a higher prevalence of common musculoskeletal disorders such as hip and elbow dysplasia [4,5].

Elbow dysplasia is an inherited condition that includes developmental anomalies such as ununited anconeal process, fragmented medial coronoid process, osteochondrosis or osteochondritis dissecans and incongruity of the elbow joint causing pain, forelimb lameness and reluctance to extend or flex the elbow joint [6].

Hip dysplasia is a multifactorial inherited condition that causes laxity of the coxo-femoral joint, subluxation and alteration of the femoral head and acetabulum resulting in abnormal joint wear and irreversible osteoarthritis, bone spurs, and degenerative joint disease causing pain, hind limb lameness and abnormal movement pattern of the limbs [7]. Latest literature shows that genetic selection based on phenotypic radiographic evidence of hip dysplasia to identify dogs for breeding less affected by the disease can help in reducing its prevalence [8]. There is also an estimated risk ratio of a dog to present both elbow and hip dysplasia simultaneously of 1.67 [9,10] suggesting that in some breeds and some cases reducing the prevalence of one of the conditions could help in reducing the prevalence of the other [7].

Some of the breeds most commonly affected by elbow and hip dysplasia include the German shepherd dog (GSD) and the Labrador retriever dog (LRD) [4,5,11–14], with musculoskeletal disorders being the most common cause of death in the GSD [11]. It is currently known that both conditions are inherited, and that larger breeds are typically more prone to express these conditions due to either genetic ancestry or conformational morphology [7].

Conformation measures and resulting movement patterns have been previously reported by Fischer and Lilje, 2014, describing the range of motion of joints in the sagittal plane and recording the stride parameters of 30 different breeds including the GSD [1]. Their study showed that each breed would typically have characteristic conformations and patterns of movement. A number of studies have characterised LRDs’ foot kinetics and/or stride parameters [15–17] and in comparison to other breeds including the Greyhound and the Rottweiler [2,18,19]. In the study by Bertram et al, 2000 [2], it was found that Greyhounds used fewer and longer strides compared to LRDs, enabling both breeds to move at a comparable relative speed. Other studies reported selected kinematic parameters [20,21] and kinematics in relation to morphology in LRDs compared to Rottweilers [22]. The latter showed that these breeds move with similar kinematic patterns, but there are differences in the magnitude of displacement and movement of the stifle and elbow joints in particular.

Other studies have characterised the GSDs kinetic or kinematics parameters in healthy [23,24] and hip dysplastic dogs [24,25]. These studies showed that the degree of hip dysplasia can affect lameness severity [25]; dysplastic dogs with no signs of lameness may still present joint kinematic alterations in the hind as well as in the forelimbs [24], and that GSDs with dysplastic hips have more extended hips during both the stance and swing phases of trot compared to their healthy counterparts [24].

To date, there are no studies comparing GSDs and LRDs nor describing any breed in standing posture. The standing position may be qualitatively described for specific breeds in the breeds standards of the respective Kennel Clubs, however, the quantification of segment and joint angles, Centre of Pressure (CoP) of the body, foot positioning and foot loadings standing has not been objectively studied. Objective descriptions of standing posture, gait and body morphology may be key in identifying links between mechanical and anatomical features. This will lead to an improved understanding of the consequences of combining features that may favour abnormal joint loading and therefore result in pathology over time.

Before we are able to infer relationships between biomechanical characteristics, conformations and clinical presentations, it is important to have baseline measures of the expected kinematic and kinetic measures in these breeds in health. This study provides a detailed quantitative description of the trotting movement and standing posture of the GSDs and LRDs by presenting kinetic and kinematic parameters for each breed, together with conformation measures. The information will also enable the identification of the main similarities and differences in the biomechanical parameters in standing and trot between two similar sized breeds.

## Materials and methods

This study was reviewed by NASPA (Non-Animal Scientific Procedures Act committee), a sub‐committee of the Animal Welfare and Ethical Review Board of the University of Surrey, before starting the trials (approval number: NASPA‐2016‐004‐SVM).

Twelve GSDs and twelve LRDs were recruited for this study, the dogs underwent a behavioural assessment and a general examination followed by a visual inspection of their gait to exclude those unfit to participate and/or lame, the assessment was carried out by a veterinary surgeon. Participants and their owners attended a session at the Animal Biomechanics Laboratory at the University of Surrey.

**Fig 1:**
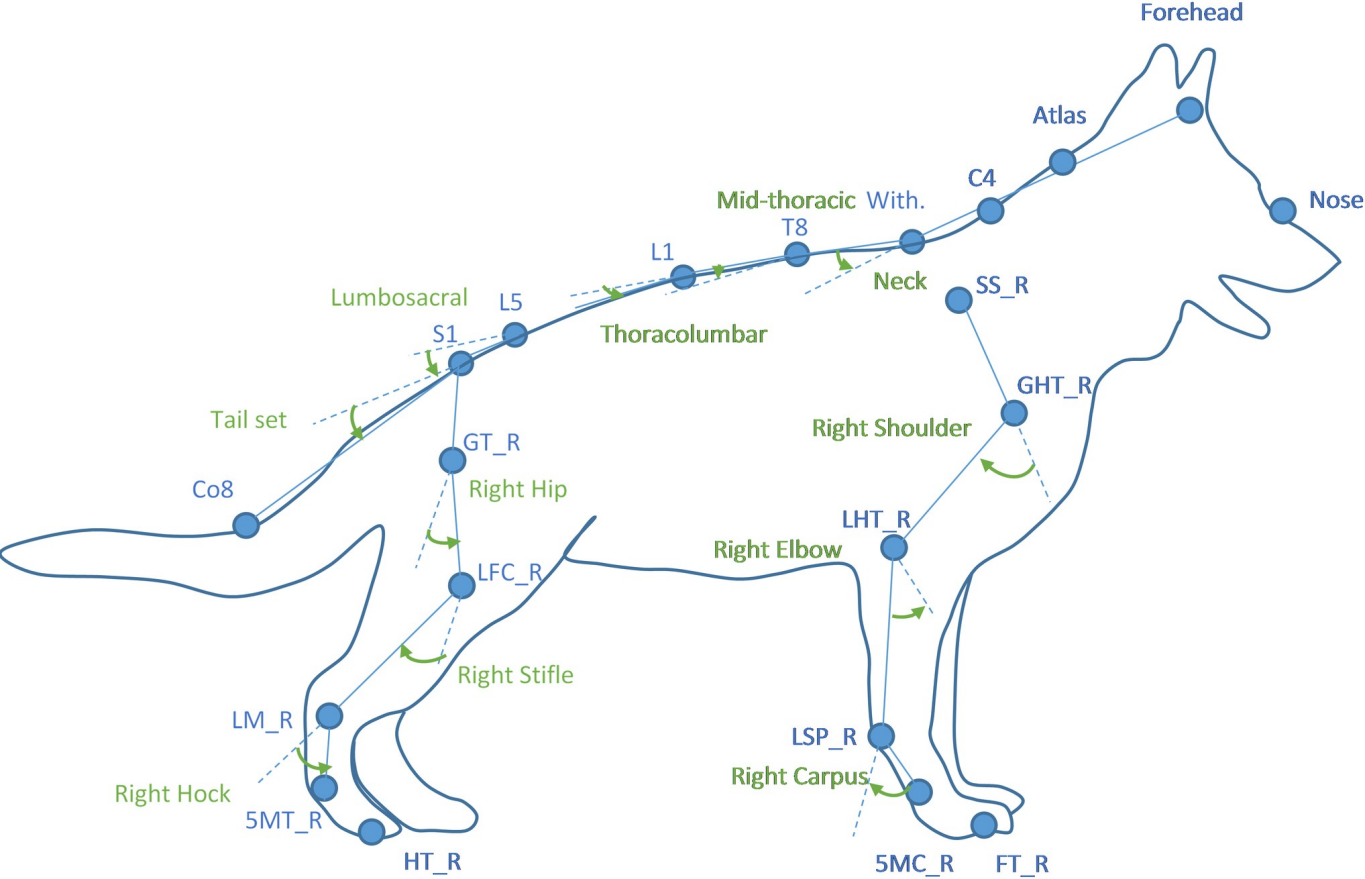
Illustration of marker positions and the markers used to compute joint rotations.

Dogs were initially familiarised with the lab and pressure walkway before their body mass was measured and the height at withers and height at sacrum were recorded using a graded measuring stick. Spherical reflective markers (9mm) were attached to the anatomical landmarks shown in Table 1 and Fig 1. The markers were used to define 2D segmental coordinate systems (see Table 1 and Fig 1), which were used to track the movement and position of segments in the sagittal and frontal planes. Hypoallergenic double-sided tape and mini hair clips were used to secure the markers to the dog’s fur after parting it with a comb.

**Table 1:** Anatomical locations for the reflective markers attached to the dogs and markers used to compute joint rotations.

Body region	Marker name	Anatomical landmark and joint represented
Head and Neck	Nose	Dorsal part of the nasal bone
	Forehead	Skull, halfway along the sagittal crest
	Atlas	Atlas
	C4	4^th^ cervical vertebra
Vertebrae centreline	With.	Withers
	T8	8^th^ thoracic vertebra
	L1	1^st^ lumbar vertebra
	L5	5^th^ lumbar vertebra
	S1	Median sacral crest
	Co8	Middle tail, 8^th^ coccygeal vertebra
Forelimbs	SS	Proximal end of the scapular spine
	GHT	Greater humeral tubercle (shoulder)
	LHT	Lateral humeral tubercle (elbow)
	LSP	Lateral styloid process (carpus)
	5MC	5^th^ metacarpophalangeal joint
	FT	2^nd^ toe (forelimb)
Hind limbs	GT	Greater trochanter (hip)
	LFC	Lateral femoral condyle (stifle)
	LM	Lateral malleolus (hock)
	5MT	5^th^ metacarpophalangeal joint
	HT	2^nd^ toe (hind limb)
Joints		Markers
Neck		Head-With.-T8
Mid-thoracic		With.-T8-L1
Thoracolumbar		T8-L1-L5
Lumbosacral		L1-L5-S1
Tail set		L5-S1-Co8
Shoulder		SS-GHT-LHT
Elbow		GHT-LHT-LSP
Carpal		LHT-LSP-5MC
Hip		S1-GT-LFC
Stifle		GT-LFC-LM
Hock		LFC-LM-5MT

Reflective marker positions were tracked using an 8-camera motion capture system (7+ Mocap, Qualisys, Gothenburg) running at 150 Hz. An approximate volume of 7x2.5x2 m was calibrated and the calibration residual was kept under 1mm for all cameras. The set up was used to record the dogs in standing posture and trot. Dogs trotted across a high resolution two and a half metre pressure sensitive walkway (7101HL, Tekscan, Biosense Medical, Chelmsford) running at 60 Hz, with 50, 688 sensors of 3.3 mm^2^ and a maximum pressure of 862 kPa. The walkway was calibrated according to the manufacturer’s instructions using a step calibration and the body mass of one of the researchers (67 kg), a real time check was then performed by walking across the walkway to ensure consistency in the amplitudes and duration of the forces. The walkway was used to record the pressures and contact areas at the metatarsal/metacarpal and digital pads of each paw for an average of three consecutive strides for each pass over the walkway. Kinetic data were recorded in synchronisation with kinematic data and video recordings. After trotting for ten passes at each dog’s comfortable speed, they stood square on the walkway three times for 10 seconds while kinematic and kinetic data were recorded. The video images were used to assess if the trials were valid. The trials were judged as valid if the dog was standing square and looking forward and in trot, if the dog was looking forward, did not trip or change speed. Trials that did not pass the validity check were discarded.

Kinetic parameters consisted of limb load distribution, total body and individual paw’s CoP trajectory and stride parameters including length, duration and speed. In addition, peak vertical forces (maximum force recorded in 2x2 sensels for the duration of stance), vertical forces (sum of all forces for the entire stance phase) and contact areas (area of loaded sensels for the entire stance phase) were calculated for each paw and for different areas of the paws i.e. digital pads vs. metacarpal/metatarsal pads. Conformational measures consisted of linear lengths of each bone calculated using the position of consecutive anatomical markers. Angular rotations were calculated in the sagittal plane using the position of three consecutive anatomical markers for the following joints: neck, mid-thoracic, thoracolumbar, lumbosacral, tail set, left and right hips, stifles, hocks, shoulders, elbows and carpi. An illustration of the calculated joint angles and the markers used to compute them is shown in Fig 1 and Table 1.

Kinetic measures involving forces were normalised to the weight of the dog by dividing the parameters by the weight as measured by the pressure mat, contact areas were divided by the body mass. Whilst stride length and CoP ranges (anterior-posterior (AP) and medial-lateral (ML)) were normalised to the size of the dog by dividing the parameters by the withers height, as recommended by Voss et al, 2011 [19].The following equations were used for the normalised dimensionless speed and stride time:

For relative speed

$$*S=S/{\left(g.WH\right)}^{1/2}$$ (Eq 1)

Where *S is the normalised speed, S is the speed, g is the gravitational acceleration and WH is the withers height.

For normalised stride time

$$ST*=ST/{\left(WH/g\right)}^{1/2}$$ (Eq 2)

Where ST* is the normalised stride time and ST is the stride time.

Data collected during trot were split into stride cycles, starting and ending with the initial contact of the left forelimb, where the time of each initial contact was determined from the pressure mat recordings. Time normalisation involved calculating and presenting continuous variables (joint rotations) at one percent increments from 0–100% of a stride cycle [24].

For each dog, 3 standing recordings and 12 trot stride cycles were used to calculate the means and standard deviations of the kinetic and kinematic parameters. Mann-Whitney tests were used to test for differences between LRDs and GSDs in the parameters describing posture and trotting movement, with significance set at p<0.05.

## Results

A total of twenty four dogs were recruited; twelve sound (lameness free) GSDs (5 males and 7 females) with a mean age of four years and one month (SD: two years and six months) and an average body mass of 36.2kg (SD: 4.1kg) and twelve sound LRDs (6 male and 6 female) with a mean age of three years and eight months (SD: two years and one month) and an average body mass of 29.3kg (SD: 4.5kg).

### Kinetic parameters in standing and trotting

Limb loads showed that the LRDs had a greater percentage weight bearing in their forelimbs compared to the GSDs (p<0.001) while standing. The LRDs carried on average 69.4% (SD: 5.0%) of their weight in their forelimbs, whereas the GSDs carried 62.4% (SD: 2.4%). Furthermore, the LRDs were shown to have a greater vertical force (p<0.001), greater peak vertical force (p = 0.001) and a greater contact area (p<0.001) in the digital pads of the forelimbs compared to the GSDs (see S1 Table). On the other hand, the metacarpal pads had a greater vertical force (Right p = 0.10, Left p<0.001) and a greater contact area (Right p = 0.028, Left p = 0.002) in the GSD.

**Fig 2:**
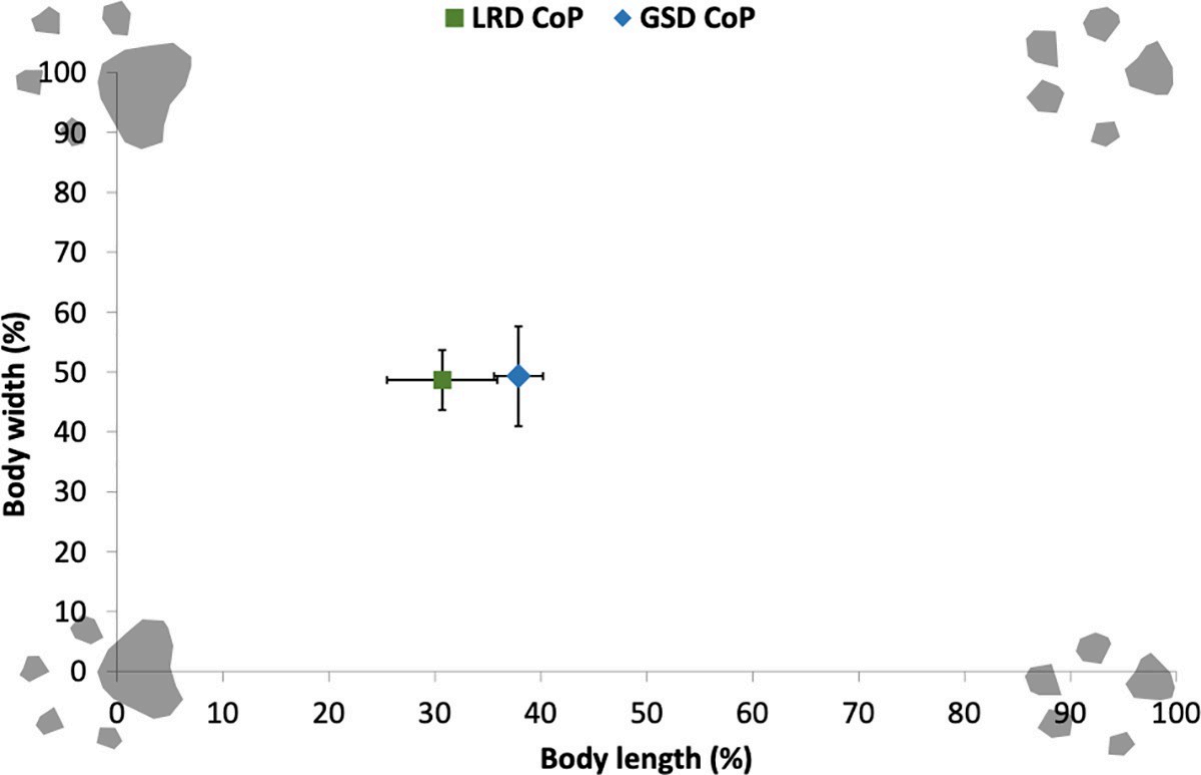
The mean location and SD of the Centre of Pressure (CoP) as a percentage of the body length and width for the LRDs and GSDs during standing in the anterior-posterior (AP) and left-right directions.

The CoP of the LRDs during standing was positioned more anteriorly (i.e. cranially) than the GSDs (p<0.001). The body CoP of the LRDs were positioned at 30.7% (SD: 5.2%) of the body length measured from the forelimbs, compared to 37.9% (SD: 2.3%) for the GSDs (Fig 2). There was no difference in the CoP location in the left-right direction (p = 0.932), as both breeds maintained a symmetrical left-right limb loading.

During trot, the LRDs had a mean vertical force in the digital pads of the forelimbs of 74. 5%BW (SD: 12.3%BW) and a mean peak vertical force in the digital pads of their forelimbs of 14.5%BW (SD: 3.0%BW, these were greater (Right p = 0.014, 0.012 and Left p = 0.003, <0.001, respectively) than those of the GSDs (58.7%BW (SD:11.5%BW) and 11.4%BW (SD:1.5%BW), respectively). The vertical force and peak vertical forces on the metacarpal pads of the forelimbs on the other hand were higher in the GSDs (Right p = 0.024, 0.039 and Left p = 0.002, 0.020, respectively). Interestingly, the changes in the distribution of the force between the digital and metacarpal pads did not result in an overall change in the forelimb force in trot (Right p = 0.887, Left p = 0.843).

**Fig 3:**
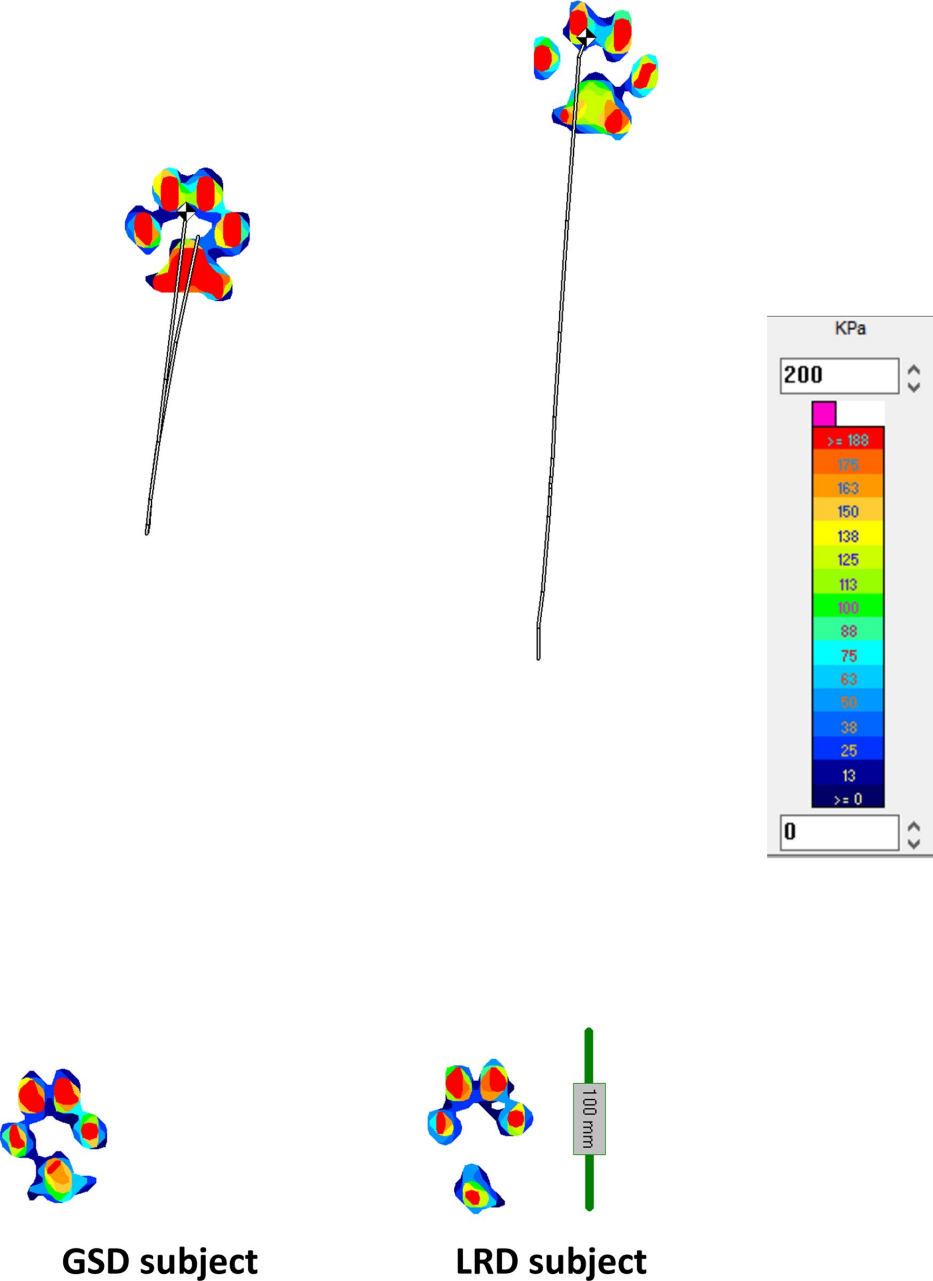
Centre of pressure (CoP) trajectory during the right diagonal stance phase of a trot stride cycle. The trajectories are typical examples from one LRD (34.2 kg, WH = 55cm) and one GSD (26.5 kg, WH = 59.5cm) trotting at similar relative speeds of 0.21. Note that considerable variability is found between and within breeds, mean values of the movement of CoP trajectories are presented in S2 Table.

**Fig 4:**
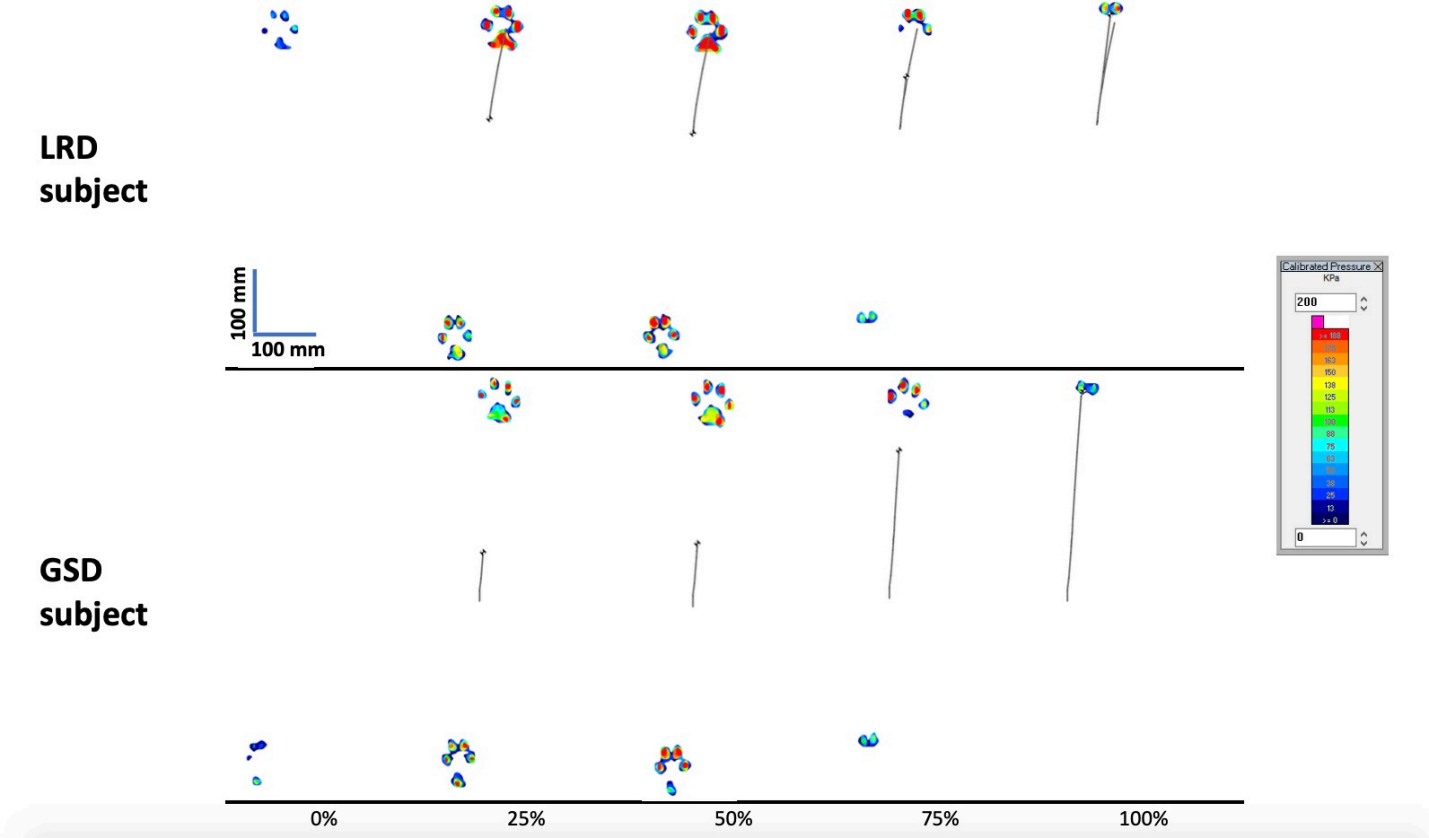
Showing limb loading, Centre of pressure (CoP) and the trajectory of the CoP during the stance phase of a right diagonal in trot. The images are at 0, 25%, 50%, 75% and 100% of a stance phase of a single stride obtained from one representative LRD (34.2 kg, WH = 55cm) and one representative GSD (26.5 kg, WH = 59.5cm) trotting at similar relative speeds of 0.21. Note that considerable variability is found between and within breeds, mean values of the movement of CoP trajectories are presented in S2 Table.

The AP range of CoP trajectory during the trot stride cycle also showed differences between the breeds (S2 Table), where the GSDs had a longer (p<0.001) AP range of 151.2%WH (SD:22.3%WH) compared to 93.4%WH (SD:24.7%WH) of the LRD. No difference was found in the left-right CoP range during trot between the breeds (p = 0.198). The difference in the movement of CoP seems to be related to the patterns of limb loading of the stance forelimb and hind limb in both breeds, whilst the LRDs load the forelimb for 99% (SD: 2%)of the stance phase, the GSDs load the forelimb for 94% (SD: 4%) (p = 0.001). The GSDs mostly load the hind limb before loading the forelimb, whilst the LRDs load the forelimb first or both limbs at the same time resulting in a shorter movement of the CoP in the AP direction. Fig 3 shows typical examples from both breeds of the movement of CoP in the stance phase of trot, and Fig 4 shows typical examples of the movement of CoP at different times during the stance phase in the two breeds.

**Fig 5:**
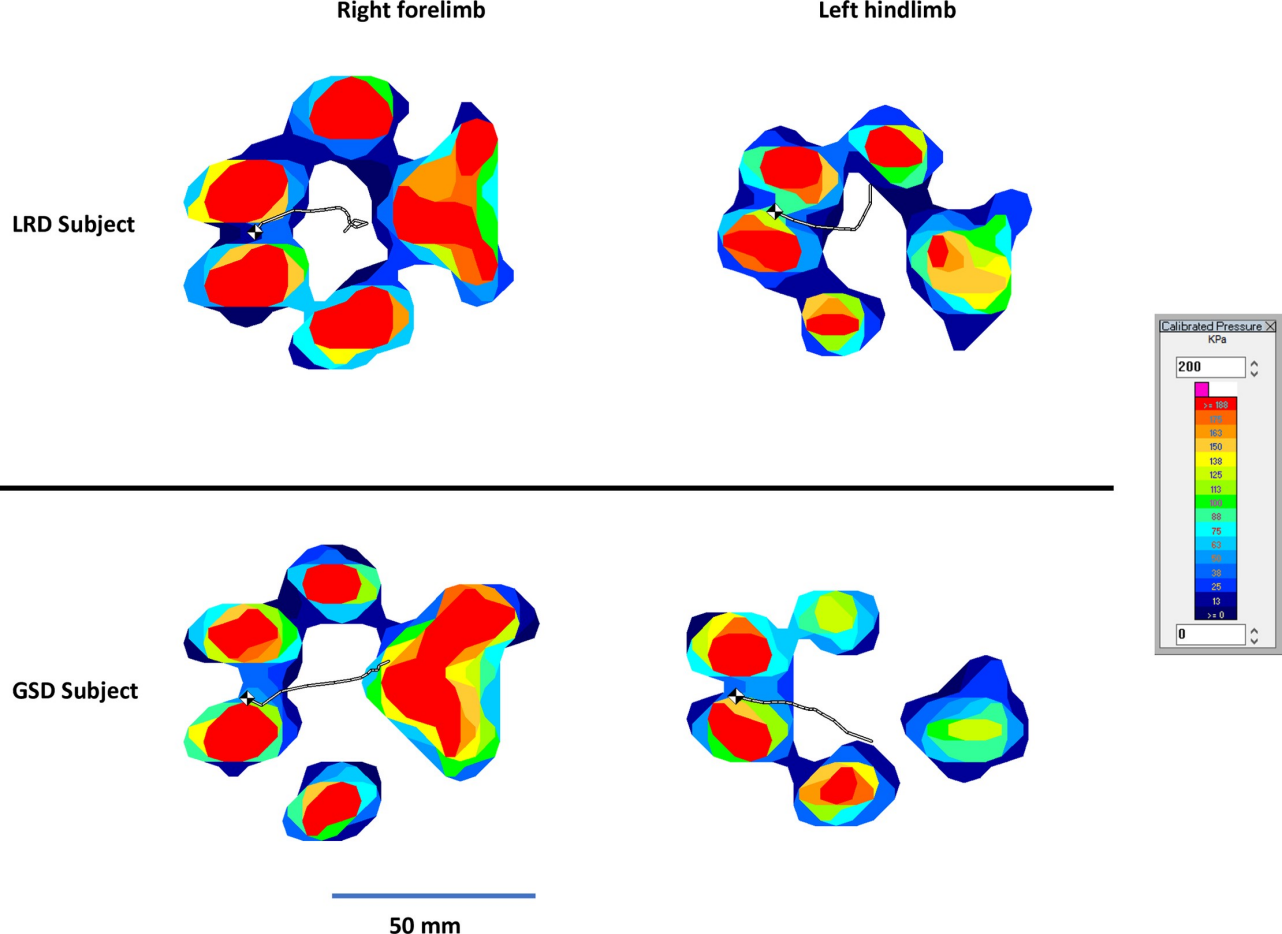
Centre of pressure (CoP) trajectory in the fore right and hind left paws during the right diagonal stance phase. The trajectories and loading patterns are examples from one LRD (34.2 kg, WH = 55cm) and one GSD (31.2 kg, WH = 60cm). Note that considerable variability is found between and within breeds and between strides, mean values of the movement of intra-paw CoP trajectories are presented in S2 Table.

Similarly, there were differences in the AP and ML ranges of the intra-paw CoP trajectory. The ranges of movement in the AP and ML directions in the forelimb (p<0.001) and hind limb (AP range p<0.001, ML range p = 0.014) in stance were greater in the GSDs compared to the LRDs (S2 Table). The AP range (forelimb and hind limb) for the GSDs ranged between 13–16% WH compared to 8–10%WH for the LRD, whilst the ML range for the GSDs was between 4–5% WH compared to 3%WH for the LRD. Fig 5 shows typical examples of the movement of the intra-paw CoP in the two breeds.

Stride parameters showed that there was no difference in the stance and swing durations of the LRDs and GSDs (p = 0.713 and p = 0.843), when these parameters are expressed as a percentage of the stride time. Similarly, the GSDs and LRDs showed no difference in their stride length (Right p = 0.378, Left p = 0.198) when normalised to the withers height [2] and there was no difference in their relative speed of trot (p = 0.755), although there was a difference in the absolute speed (LRDs: 2.19m/s vs. GSDs: 2.45m/s, p = 0.012) as shown in S3 Table.

### Kinematic parameters during standing and trotting

**Fig 6:**
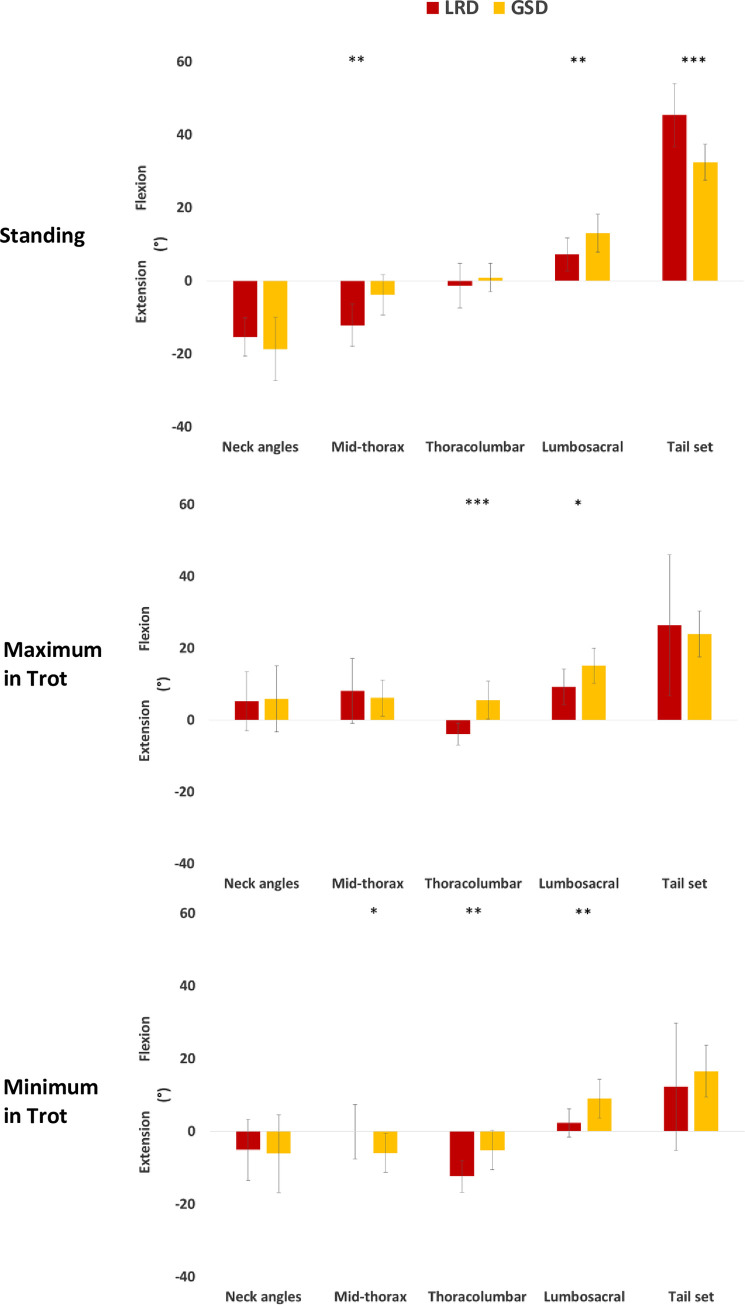
Plots of the mean and standard deviations of the flexion/extension angles of the back during standing, and the maximum and minimum values during trot for the LRDs and GSDs. Significant differences are denoted by a * for p<0.05, ** for p<0.01 and *** for p<0.001.

**Fig 7:**
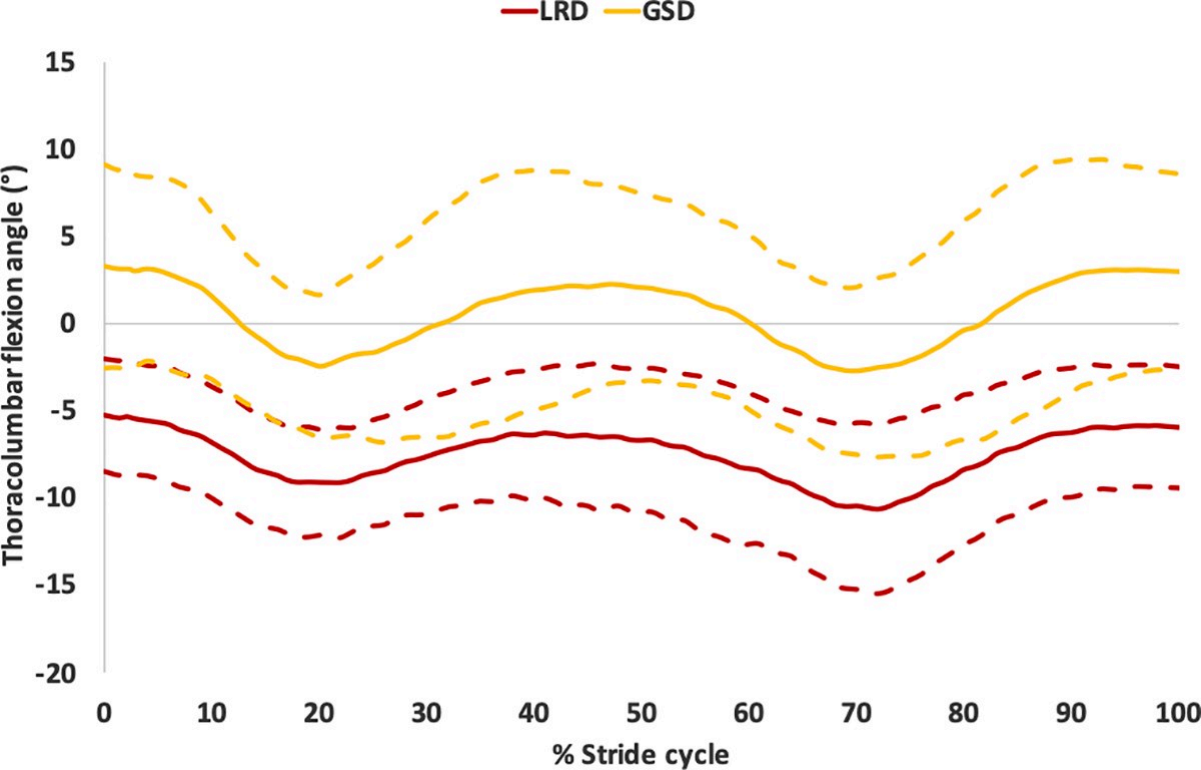
Showing the means (solid lines) and +/- 1 standard deviations (dashed lines) of the flexion angle of the thoracolumbar joint during the trot stride cycle for the LRDs and GSDs.

The rotations of the angles of the back varied between the breeds during standing and trot. The LRDs had a more extended thoracic angle (12.2°, SD: 5.8°) compared to the GSDs (3.8°, SD: 5.6°) during standing (p = 0.002), as shown in Fig 6. The LRDs also had a more flexed tail carriage angle while standing (p<0.001). However, during trot there was no difference in the tail angle between breeds. During trot, the LRDs had a more extended thoracolumbar joint angle (maximum and minimum p<0.001) and a less flexed lumbosacral angle (maximum p = 0.017 and minimum p = 0.002) throughout the trot stride cycle (Fig 7). The mean flexion angles of the back joints for each breed are shown in S4 Table.

**Fig 8:**
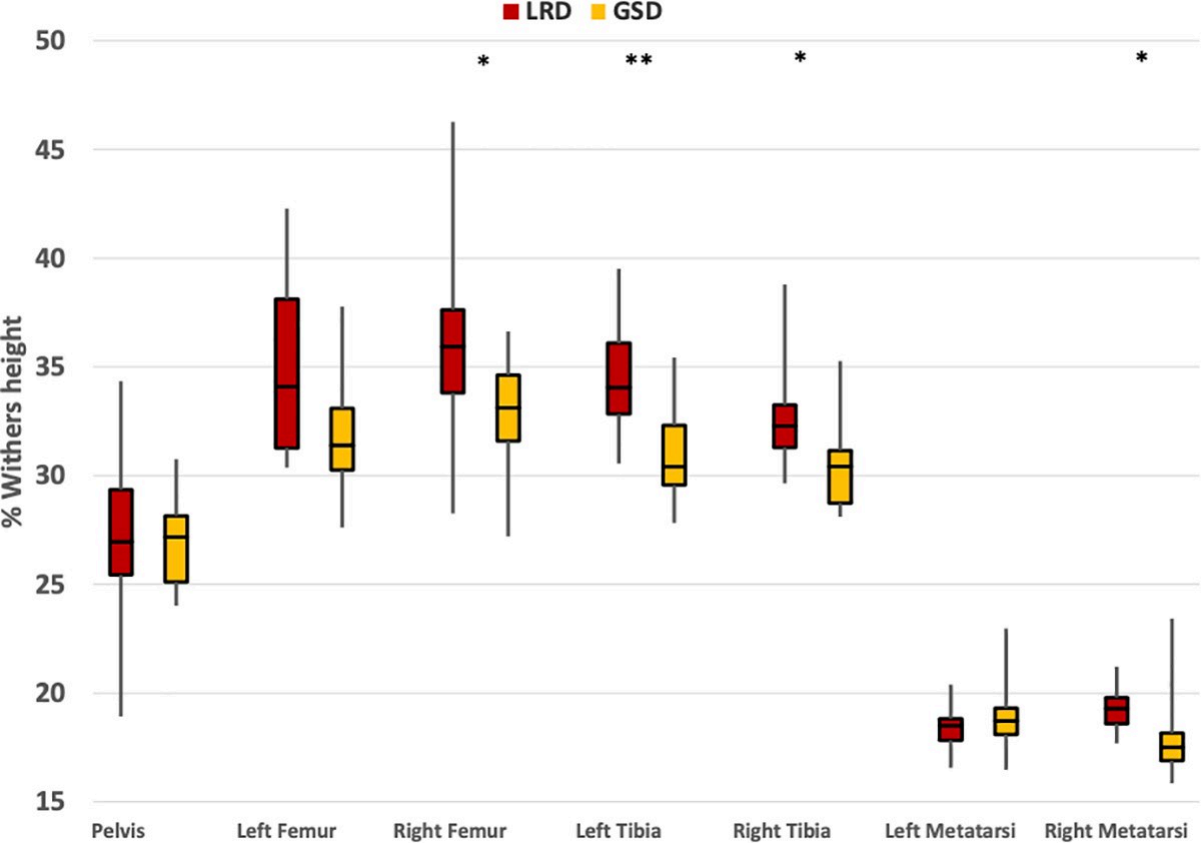
Length of the bones in the hind limb, as a percentage of the height at withers, showing the mean for the breed and standard deviations. Significant differences are denoted by an (* for p<0.05, ** for p<0.01).

The mean length of the bones and segments for each breed, measured using the anatomical landmarks of the bones are shown in S5 Table and are expressed as a percentage of the height at the withers. A summary of the findings is given here, differences are only reported when they are found in both the right and left limbs. In the hind limb, the tibia bones were shorter in the GSDs with a mean of 30.7%WH ± 2.3%WH compared to 33.6%WH± 2.8%WH in the LRDs (Right p = 0.021, Left p = 0.005), expressed as a percentage of the withers height (Fig 8). In the forelimb, the GSDs had a shorter (Right p = 0.002, Left p = 0.001) humerus (26.04%, SD: 3.01%) and shorter (Right p = 0.002, Left p = 0.010) radius (34.40%, SD: 1.72%) compared to the LRDs (30.52%, SD: 2.40%; 36.95%, SD: 2.37%, respectively). There was no difference in the length of the metacarpal bones. Furthermore, there was a difference in the length of the lumbar region (p = 0.028) and the back length measured from the withers to the sacrum (p = 0.008), where the GSDs had means of 20.4% (SD: 3.0%WH) and 86.1%WH (SD: 7.6%) compared to 17.8%WH (SD: 3.4%WH) and 77.8%WH (SD:5.0%WH) in the LRDs of the lumbar region and the full back respectively.

While standing, the hock and stifle flexion angles were greater (hock: Right = 0.007, Left<0.001; stifle: Right = 0.005, Left = 0.028) for the GSDs (57.5°, SD: 10.8° and 44.2°, SD: 8.4°) than the LRDs (26.3°, SD: 9.1° and 31.5°, SD: 15.3°, respectively) but the hip joint was more flexed (Right and Left p<0.001) in the LRDs (29.6°, SD: 12.2°), whereas the GSDs hip was more extended (-9.6°, SD: 10.7°) (S4 Table). In the forelimbs, there was no difference in the flexion angles of both elbows or carpal joints, but shoulder flexion was greater (Right p<0.001, Left p = 0.039) in the LRDs with a mean of 73.2° (SD: 8.5°) compared to 59.8° (SD: 9.0) in the GSDs as shown in S4 Table.

**Fig 9:**
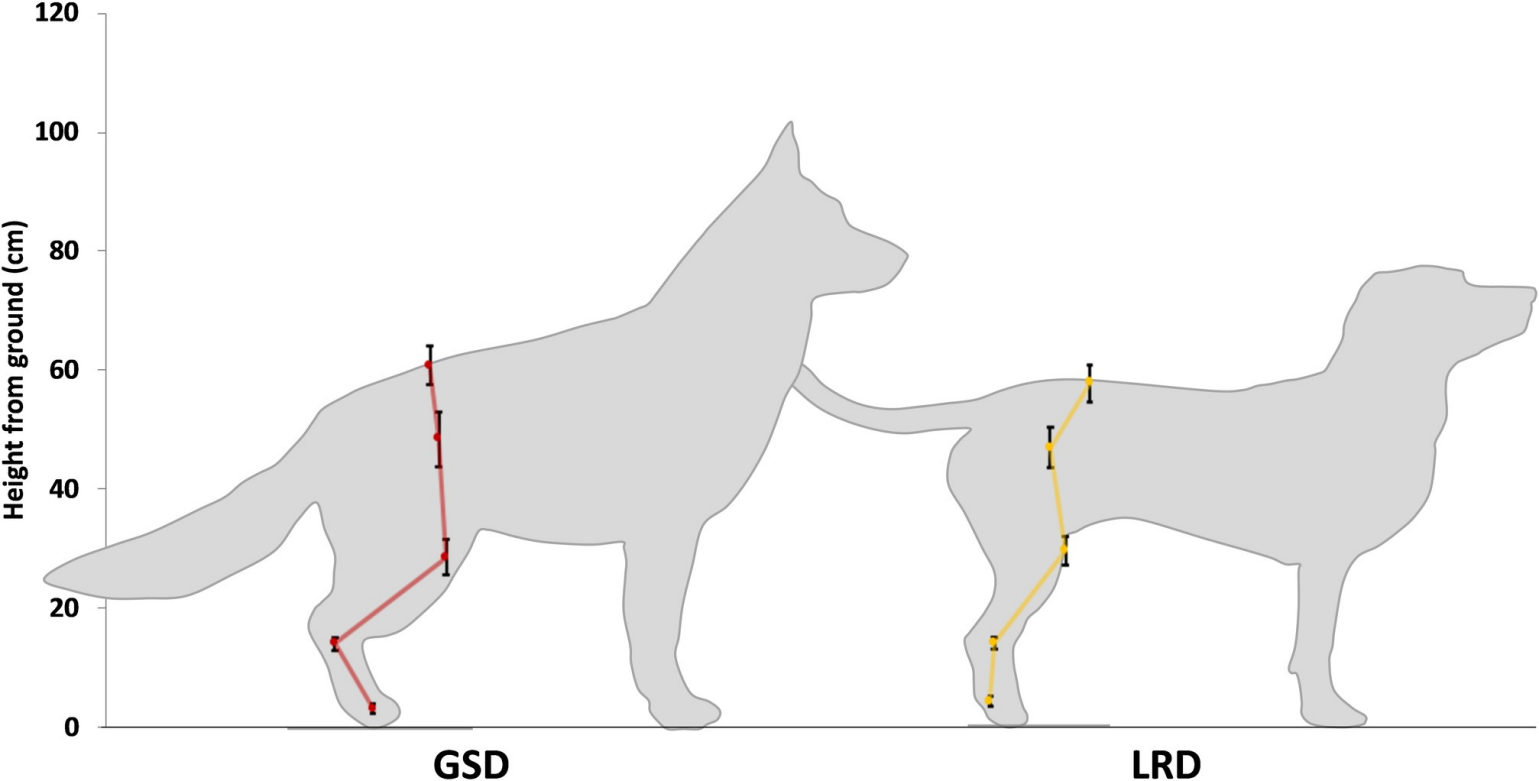
A sketch showing the height of the anatomical landmarks of the hind limb above ground for the GSDs and LRDs. Each point shows the mean height above ground for the breed and the standard deviation.

The greater flexion in the GSD’s lumbosacral joint of the back resulted in a greater incline of the lumbar spine and an increased tilt of the pelvis (p = 0.004) when measured with respect to the horizontal (78.8°, SD: 7.9°) compared to the LRDs (66.2°, SD: 9.3°). The more flexed hocks and stifles, extended hips and shorter tibias in the GSDs’ hind limb compared to the LRDs meant that the sacrum and the anatomical landmarks of the hind limb in the GSDs are closer to the ground, this is demonstrated in Fig 9.

**Fig 10:**
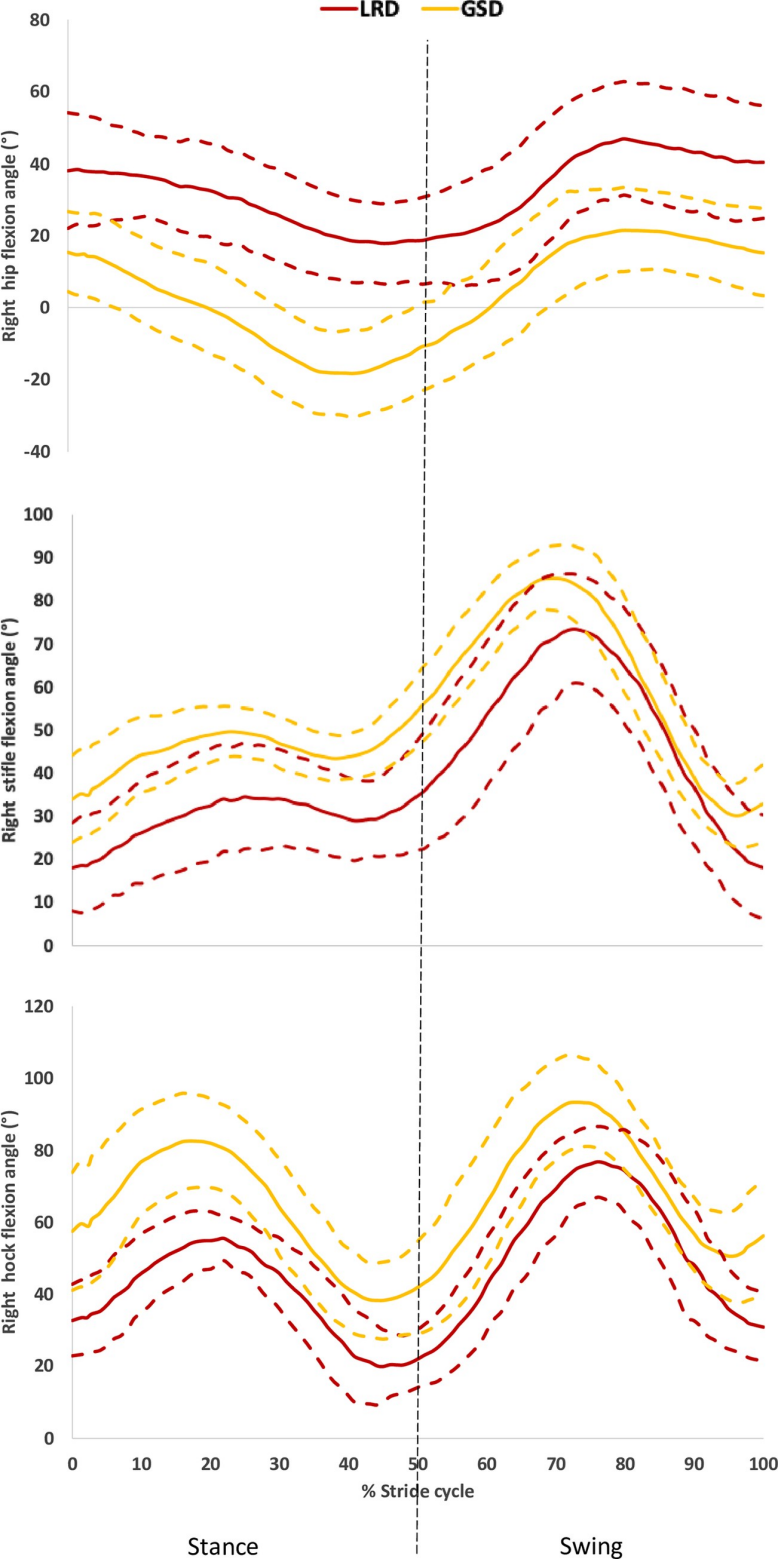
Plots of the flexion angle of the left hip (a), left stifle (b) and left hock (c) during trot, as a percentage of the stride cycle. The solid line shows the mean for each breed and the dashed lines show one standard deviation from the average.

During trot, the thoracolumbar joint was more flexed in the GSD, whereas it was always extended throughout the stride cycle in the LRDs (maximum and minimum p<0.001). The lumbosacral joint was flexed throughout the stride cycle in both breeds, where the GSDs had a greater flexion (maximum p = 0.017 and minimum p = 0.002). The maximum and minimum flexion angles of the joints of the back during trot are shown in S4 Table. In the hind limb, the flexion/extension angles during trot also differed between the breeds, as shown in S4 Table and Fig 10, the LRD’s hips remained flexed during the trot stride cycle, whereas the GSDs hip joints were less flexed during swing phase (Right and Left maxima p<0.001), and more extended during the later stages of the stance phase and the beginning of the swing phase (Right and Left minima p<0.001). The stifle was always flexed in both breeds during the stance and swing phases although the flexion angle was greater (Right maxima p = 0.028, Left maxima p = 0.033 and Right minima p = 0.001, Left minima p = 0.024) in the GSDs (maximum mean of 88.9°, SD: 8.0° and minimum mean 27.8°, SD: 8.4°) compared to the LRDs (maximum mean 79.4°, SD: 10.8° and minimum mean 15.5°, SD: 10.7°). GSDs had greater maximum flexion of the hock compared to LRDs (Right p = 0.004, Left p = 0.005) and this was more pronounced during the stance phase as shown in Fig 10 for the left limb. In contrast, there were no differences in the flexion/extension angles of the joints in the forelimb during stance and swing phase of the trot stride cycle (S4 Table).

## Discussion

The two large canine breeds investigated in this study are breeds with high prevalence of common musculoskeletal disorders such as hip and elbow dysplasia, as reported in the literature [4,5,11–14,26]. Such disorders may be linked to conformational and biomechanical features that could cause overloading of the joints and result in clinical problems. Therefore, the aim of this study was to investigate both conformation and biomechanical parameters in the standing posture and during trot in non-lame subjects of these two breeds. The results showed similarities in their stride parameters (after normalisation), but the majority of the biomechanical parameters investigated, particularly the distribution of forces in the forelimb, measures of the CoP in the stance of the limbs and within paws and the vertebral column and hind limbs joint rotations were different between the two breeds.

The kinetic analyses showed that the Labradors bear considerably more weight in their forelimbs compared to the German shepherds during standing position, resulting in their body CoP to be located more anteriorly i.e. cranially. It should be noted that, as far as the authors knowledge, the body CoP in standing position and its trajectory during trot has not been reported in dogs, there is therefore no information available in other breeds to make direct comparisons, and the implications of these differences are unknown. Although it is known that dogs support around 60% of their weight in the forelimbs due to the weight of the head, neck and thorax [27], the LRD’s forelimbs weight bearing of near 70% is more than in the average breed. In a study by Bertram et al, 2000, five trotting LRDs showed on average 66% of its body weight supported by the forelimbs, similarly to our study, and they concluded that this parameter was comparable between LRDs and Greyhounds [2].

In our study, a greater proportion of the weight in the LRDs in standing and trotting was supported in their digits instead of distributed over the digital and metacarpal pads as in the GSDs, resulting in higher vertical forces and peak vertical forces supported by the fore digital pads in the LRD. This differs from the results found by Besancon et al, 2004, where the peak vertical force in the forelimbs of Labrador retrievers was greater in the metacarpal pad than the digital pads, however, their study considered walking gait making it difficult to compare to the values in trot reported here [3]. In contrast, Souza et al, 2013 found that GSDs load their metacarpal pads more than the digital ones [23], which is closer to what was found in our study where loadings between the digital and metacarpal and metatarsal pads in GSDs are more evenly distributed. The discrepancies between studies may be due to a number of reasons, including speed, type of gait (walk vs. trot) and even subtle differences in the breed lines and individual genetics between animals of different continents and also differences in the technologies used (sensors, versions and resolutions). Independent of this, all studies seem to agree in that both the digital and the metacarpal/metatarsal pads play an important role in weight bearing in the dog. The results for the intra-paw ranges of CoP excursions further support this difference between breeds; the intra-paw CoP trajectory moves significantly more in the anterior-posterior and medial-lateral directions in the GSDs suggesting that the force is distributed more evenly within the paw than in the LRDs which makes more use of the digital pads in the stance phase of the forelimb. The CoP trajectories of both breeds were comparable to those described by Oosterlinck et al, 2011 [28].

Similarly, we found differences between the body CoP trajectory of both breeds in trot, being longer in the GSDs than in the LRD. The CoP trajectory is directly affected by the braking (fore mainly) and pushing force (hind mainly) of the limbs [1]. In both breeds, fore and hind limbs are weight bearing for the majority of the stance phase, as also shown by Fischer and Lilje, 2014 [1], but there seems to be differences in the amount of use of fore and hind limbs at the beginning and end of the stance phase between these breeds which results in a considerable difference in the movement of the CoP during the stance phase. This may be an indication that the GSDs is comfortable in moving the weight considerably between the hind and forelimb, while the LRDs relies more in the forelimbs than on the hind limbs for stance support.

Kinematic analyses during standing showed that the pelvis of the GSDs was more tilted than the LRDs, together with a more flexed lumbosacral joint angle, more extended hips and more flexed stifles and hocks. This was accompanied by shorter tibial segments, resulting in a reduced hind limb height in the GSDs. The lengths of the bone segments were similar to those found in the Jena study by Fischer and Lilje, 2014 in GSDs, where 10 GSDs from a working background and 14 GSDs with a background in the show ring were studied, except that they found an even shorter femur in the show line GSDs [1]. Our GSDs were not classified by their breed line as the number of dogs would have not allowed enough statistical power.

During trot, the LRDs maintained their hips in a slightly flexed position for the whole stride, in contrast to the GSD’s hip joints, which were less flexed at the start of the stance phase and more extended at the end of the stance phase compared to the LRDs. The hips extension of the GSDs also necessarily results in more flexed stifles and hocks during stance phase. Miqueleto et al, 2013 found that the hips of healthy GSDs had a maximum flexion angle of 38° during trot, whereas GSDs with hip dysplasia had a maximum hip flexion angle of 31° [24]. This suggests that dogs with more extended hips might be more susceptible to hip dysplasia or that hip dysplasia results in more extended hips. There is no equivalent biomechanical study of Labradors with hip dysplasia to compare with. However, recent studies show that the prevalence and severity of hip dysplasia may be reduced by phenotypic screening of joint conformation as a strategy for breeders to make selection decisions [7,29].

To calculate the mean of the kinetic parameters and to enable comparisons to be made between individuals and between the breeds, kinetic parameters were expressed as a percentage of body weight, contact areas were divided by the body mass and the stride parameters (speed and stride duration) and ranges of CoP trajectories were normalised to the withers height. Other studies have also normalised force to body weight and compared parameters at the same relative speed [2,19]. Similarly, distances measured from kinematic recordings such as the length of the bones and height of the joints above ground were normalised to the size of the dog and expressed as a percentage of the height at withers [2]. These normalisations allow a comparison between breeds to be made, however, they are not without limitations. For example, normalisations to the withers height assume that it is a comparable measure of dog size across breeds, but in reality this is likely to introduce certain biases to spatial measures. However, other parameters that may be used to normalise for size will have similar limitations.

Dogs were allowed to trot at their normal, comfortable speed and stand square but comfortably, during data collection. This approach has also been used by others to ensure the handler has no influence on the posture or speed of trot [2,30,31]. Collecting repeat recordings of each dog’s trot and posture showed that dogs trotted consistently at their comfortable speed with fairly low intra-subject variation (mean 0.15 m/s, 0.64 relative speed) and they were consistent in their movement and posture as the intra-subject variations for other kinematic (typically between 3–5°) and kinetic parameters (typically 3–9%BW) were also small. However, this variability between strides and between dogs of the same breed together with the limited sample sizes used in biomechanical studies can have an effect on the results. Similarly, the kinematic data presented here is limited by errors caused by skin and fur artefact and use of 2D marker system that does not take into account bone axial rotations.

In our study we found that GSDs stand with increased incline of the pelvis and a more flexed thoracic and lumbosacral joint than LRDs. The GSD breed is known to be more susceptible to lumbosacral disease [32], however, currently, there is no evidence to suggest that these two findings may be linked. In a similar way, we found that LRDs have a greater forelimb weight bearing than the GSDs, while they present a higher risk for shoulder osteochondrosis [12]. Similarly here, there is no clear causal-effect relationship between the presentation of the disorder and the biomechanical findings observed. Therefore, further research in animals affected by musculoskeletal disorders will be required to investigate if conformation, posture and movement relate to specific musculoskeletal disorders within these breeds.

In conclusion, this study provided detailed description of posture and movement of the LRDs and GSDs and identified similarities and differences between these two large breeds who are at higher risk of developing specific musculoskeletal disorders. The increased weight bearing in the LRDs’ forelimbs during standing and the more exaggerated hip extension and stifle and hock flexion angles of the GSDs may place increased strain on their joints; hence it could be speculated that they may be more likely to develop musculoskeletal conditions in these regions. Further investigation in sound and affected animals would be needed to establish such relationships, including inverse dynamics of the hind limbs, as well as studying show and working lines that present different conformations within the GSDs breed. Consequently, this baseline of normal movement of the LRDs and GSDs provides a detailed description for comparisons in future studies focussing on breeding strategies and presentations of musculoskeletal disorders in these two breeds.

## Supporting information

S1 Table The mean and standard deviation of kinetic parameters for the LRDs and GSDs during standing and trotting. P values less than 0.05 are in bold. RF: Right fore, LF: Left fore, RH: Right hind, LH: Left hind, %BW: Percentage of body weight.
(DOCX)

S2 Table Durations of weight bearing of the stance limbs as a percentage of the stance phase, the ranges of the movement of the CoP trajectory in a stance phase in the anterior-posterior (AP) direction and left-right direction, and the ranges of the CoP trajectory within the paw (intra-paw) in the AP and medial-lateral (ML) directions for LRD and GSD. The spatial parameters are presented as a percentage of the withers height (WH). P values less than 0.05 are in bold. RF: Right fore, LF: Left fore, RH: Right hind, LH: Left hind.
(DOCX)

S3 Table The mean and SD values for stride parameters for the LRDs and GSDs during trotting. P values less than 0.05 are in bold. The spatial parameters are presented as a percentage of the withers height (WH).
(DOCX)

S4 Table The mean maximum and minimum values for flexion angles in the sagittal plane for the LRDs and GSDs during standing and trotting. P values less than 0.05 are in bold.
(DOCX)

S5 Table Bone and segment linear lengths of the LRDs and GSDs. Expressed as a percentage of the height at withers, which were measured using the positions of consecutive anatomical markers. P values less than 0.05 are highlighted in bold. For markers’ names see Table 1 in the manuscript.
(DOCX)
