# Supplementary material for: Biomechanical comparison of standing posture and during trot between German shepherd and Labrador retriever dogs
Source: PLoS One. 2020 Oct 2;15(10):e0239832. doi: 10.1371/journal.pone.0239832 (PMC7531786; doi:10.1371/journal.pone.0239832)
Supplement: S1 Table — P values less than 0.05 are in bold. RF: Right fore, LF: Left fore, RH: Right hind, LH: Left hind, %BW: Percentage of body weight. (DOCX) [file pone.0239832.s001.docx]

**Supplementary material:**

**Table S1: The mean and standard deviation of kinetic parameters for the LRDs and GSDs during standing and trotting.** P values less than 0.05 are in bold. RF: Right fore, LF: Left fore, RH: Right hind, LH: Left hind, %BW: Percentage of body weight.

|  |  | **Standing** | | | | | **Trotting** | | | | |
| --- | --- | --- | --- | --- | --- | --- | --- | --- | --- | --- | --- |
| **Kinetic parameter** | **Limb** | **LRD** | | **GSD** | |  | **LRD** | | **GSD** | |  |
|  |  | **Mean** | **SD** | **Mean** | **SD** | **P value** | **Mean** | **SD** | **Mean** | **SD** | **P value** |
| Forelimb-Hind limb weight bearing (% Weight Bearing) | Fore | 69.40 | 4.99 | 62.37 | 2.43 | **<0.001** | 63.10 | 1.50 | 62.34 | 2.06 | 0.410 |
|  | Hind | 30.60 | 4.99 | 37.63 | 2.43 | **<0.001** | 36.90 | 1.50 | 37.66 | 2.06 | 0.410 |
| Left-Right weight (% Weight Bearing) | Right | 49.86 | 4.33 | 50.20 | 3.35 | 0.977 |  | | | | |
|  | Left | 50.14 | 4.33 | 49.80 | 3.35 | 1.000 |  | | | | |
| Vertical force (%BW) | RF | 34.19 | 3.90 | 28.97 | 4.87 | **0.020** | 124.06 | 19.94 | 122.79 | 20.79 | 0.887 |
|  | LF | 35.12 | 3.80 | 29.86 | 4.81 | **0.014** | 124.20 | 19.01 | 122.19 | 20.07 | 0.843 |
|  | RH | 15.54 | 3.67 | 17.98 | 2.49 | 0.114 | 72.39 | 13.21 | 73.22 | 10.11 | 0.551 |
|  | LH | 15.04 | 2.42 | 17.31 | 3.12 | 0.060 | 73.17 | 14.04 | 73.14 | 10.34 | 0.755 |
| Vertical force in the digital pads (%BW) | RF | 28.55 | 3.31 | 19.00 | 5.66 | **<0.001** | 73.68 | 11.79 | 59.24 | 11.99 | **0.014** |
|  | LF | 27.59 | 3.67 | 17.84 | 5.18 | **<0.001** | 75.28 | 12.70 | 58.20 | 11.10 | **0.003** |
|  | RH | 14.65 | 3.83 | 14.97 | 2.36 | 0.671 | 54.81 | 12.68 | 54.25 | 10.63 | 0.799 |
|  | LH | 14.71 | 2.78 | 14.36 | 2.79 | 0.755 | 55.90 | 13.08 | 54.77 | 10.02 | 0.713 |
| Vertical force in the metacarpal/tarsal pads (%BW) | RF | 5.75 | 2.58 | 9.97 | 3.71 | **0.012** | 50.37 | 12.03 | 62.55 | 10.80 | **0.024** |
|  | LF | 7.53 | 4.03 | 12.03 | 4.40 | **0.012** | 48.92 | 9.59 | 63.98 | 10.96 | **0.002** |
|  | RH | 0.88 | 1.36 | 3.00 | 2.40 | **0.010** | 17.58 | 3.98 | 18.96 | 5.65 | 0.347 |
|  | LH | 0.33 | 0.62 | 2.95 | 2.19 | **<0.001** | 17.26 | 5.03 | 18.37 | 5.44 | 0.347 |
| Peak vertical force in the digital pads (%BW) | RF | 5.60 | 0.93 | 3.95 | 1.54 | **0.001** | 14.32 | 3.04 | 11.58 | 1.81 | **0.012** |
|  | LF | 5.62 | 1.45 | 3.69 | 1.22 | **0.001** | 14.58 | 2.86 | 11.29 | 1.10 | **<0.001** |
|  | RH | 3.82 | 0.97 | 3.27 | 0.52 | 0.114 | 11.78 | 3.51 | 11.74 | 2.19 | 0.630 |
|  | LH | 4.16 | 0.71 | 3.07 | 0.55 | **<0.001** | 11.86 | 3.44 | 11.22 | 1.86 | 0.887 |
| Peak vertical force in the metacarpal/tarsal pads (%BW) | RF | 1.81 | 0.59 | 2.03 | 0.68 | 0.410 | 9.54 | 2.59 | 12.17 | 2.73 | **0.039** |
|  | LF | 2.14 | 0.74 | 2.26 | 0.62 | 0.590 | 9.50 | 2.48 | 12.25 | 2.23 | **0.020** |
|  | RH | 0.44 | 0.64 | 1.16 | 0.74 | 0.014 | 5.94 | 1.61 | 6.33 | 1.68 | 0.410 |
|  | LH | 0.21 | 0.38 | 1.15 | 0.63 | **0.001** | 5.91 | 1.52 | 6.52 | 1.78 | 0.378 |
|  |  |  |  |  |  |  |  |  |  |  |  |
| Contact area in the digital pads (cm^2^/kg) | RF | 0.36 | 0.08 | 0.26 | 0.03 | **<0.001** | 0.54 | 0.10 | 0.46 | 0.08 | 0.068 |
|  | LF | 0.35 | 0.05 | 0.25 | 0.03 | **<0.001** | 0.55 | 0.11 | 0.45 | 0.07 | **0.028** |
|  | RH | 0.24 | 0.06 | 0.23 | 0.03 | 0.630 | 0.45 | 0.09 | 0.41 | 0.06 | 0.291 |
|  | LH | 0.24 | 0.06 | 0.23 | 0.03 | 0.590 | 0.45 | 0.09 | 0.42 | 0.06 | 0.241 |
|  |  |  |  |  |  |  |  |  |  |  |  |
| Contact area in the metacarpal/tarsal pads (cm^2^/kg) | RF | 0.15 | 0.05 | 0.18 | 0.05 | 0.052 | 0.33 | 0.09 | 0.36 | 0.08 | 0.291 |
|  | LF | 0.16 | 0.04 | 0.20 | 0.04 | **0.024** | 0.34 | 0.08 | 0.37 | 0.07 | 0.143 |
|  | RH | 0.03 | 0.04 | 0.07 | 0.04 | **0.028** | 0.19 | 0.03 | 0.18 | 0.03 | 0.977 |
|  | LH | 0.01 | 0.03 | 0.07 | 0.04 | **0.002** | 0.18 | 0.04 | 0.18 | 0.04 | 0.478 |
