# Supplementary material for: Biomechanical comparison of standing posture and during trot between German shepherd and Labrador retriever dogs
Source: PLoS One. 2020 Oct 2;15(10):e0239832. doi: 10.1371/journal.pone.0239832 (PMC7531786; doi:10.1371/journal.pone.0239832)
Supplement: S2 Table — The spatial parameters are presented as a percentage of the withers height (WH). P values less than 0.05 are in bold. RF: Right fore, LF: Left fore, RH: Right hind, LH: Left hind. (DOCX) [file pone.0239832.s002.docx]

**Table S2: Durations of weight bearing of the stance limbs as a percentage of the stance phase, the ranges of the movement of the CoP trajectory in a stance phase in the anterior-posterior (AP) direction and left-right direction, and the ranges of the CoP trajectory within the paw (intra-paw) in the AP and medial-lateral (ML) directions for LRD and GSD.** The spatial parameters are presented as a percentage of the withers height (WH). P values less than 0.05 are in bold. RF: Right fore, LF: Left fore, RH: Right hind, LH: Left hind.

| Kinetic parameter | Limb | LRD | | GSD | | p-value |
| --- | --- | --- | --- | --- | --- | --- |
|  |  | Mean | SD | Mean | SD |  |
| Time of weight-bearing | RF | 99.01 | 1.93 | 94.58 | 3.72 | **0.001** |
| (% stance phase) | LF | 98.62 | 2.00 | 93.97 | 4.41 | **0.006** |
|  | Stance Forelimb | 98.82 | 1.73 | 94.28 | 3.63 | **<0.001** |
|  | RH | 83.20 | 5.37 | 86.12 | 5.76 | 0.319 |
|  | LH | 82.61 | 4.67 | 86.36 | 6.24 | 0.160 |
|  | Stance Hind limb | 82.91 | 4.45 | 86.24 | 5.78 | 0.266 |
| Stance phase CoP | | | | | | |
| AP range (%WH) | RF-LH | 89.29 | 33.35 | 152.23 | 35.40 | **<0.001** |
|  | LF-RH | 97.50 | 24.81 | 150.25 | 31.70 | **<0.001** |
|  | Stance limbs | 93.40 | 24.70 | 151.24 | 22.34 | **<0.001** |
| Left-Right range (%WH) | RF-LH | 20.39 | 8.33 | 15.09 | 5.81 | 0.052 |
|  | LF-RH | 16.57 | 7.52 | 15.56 | 5.47 | 0.932 |
|  | Stance limbs | 18.48 | 6.22 | 15.32 | 4.25 | 0.198 |
| Intra-paw CoP | | | | | | |
| AP range (%WH) | RF | 9.59 | 1.99 | 12.81 | 3.42 | **0.005** |
|  | LF | 9.47 | 2.00 | 13.87 | 2.69 | **<0.001** |
|  | Stance Forelimb | 9.53 | 1.88 | 13.34 | 2.39 | **<0.001** |
|  | RH | 7.58 | 2.35 | 14.02 | 3.87 | **<0.001** |
|  | LH | 8.19 | 2.21 | 15.27 | 4.53 | **<0.001** |
|  | Stance Hind limb | 7.88 | 1.80 | 14.64 | 2.96 | **<0.001** |
| ML range (%WH) | RF | 2.72 | 0.94 | 4.97 | 1.08 | **<0.001** |
|  | LF | 2.76 | 0.70 | 4.80 | 0.79 | **<0.001** |
|  | Stance Forelimb | 2.74 | 0.67 | 4.88 | 0.83 | **<0.001** |
|  | RH | 2.92 | 0.70 | 3.62 | 1.02 | 0.101 |
|  | LH | 2.56 | 0.74 | 3.53 | 0.70 | **0.005** |
|  | Stance Hind limb | 2.74 | 0.67 | 3.58 | 0.76 | **0.014** |
