# Supplementary material for: Biomechanical comparison of standing posture and during trot between German shepherd and Labrador retriever dogs
Source: PLoS One. 2020 Oct 2;15(10):e0239832. doi: 10.1371/journal.pone.0239832 (PMC7531786; doi:10.1371/journal.pone.0239832)
Supplement: S3 Table — P values less than 0.05 are in bold. The spatial parameters are presented as a percentage of the withers height (WH). (DOCX) [file pone.0239832.s003.docx]

**Table S3: The mean and SD values for stride parameters for the LRDs and GSDs during trotting.** P values less than 0.05 are in bold. The spatial parameters are presented as a percentage of the withers height (WH).

| Stride parameter | LRD | | GSD | |  |
| --- | --- | --- | --- | --- | --- |
|  | Mean | SD | Mean | SD | P value |
| Stance duration (% stride time) | 43.22 | 1.75 | 43.71 | 2.91 | 0.713 |
| Swing duration (% stride time) | 57.21 | 2.18 | 56.80 | 2.71 | 0.843 |
| Stride time (s) | 0.50 | 0.03 | 0.53 | 0.03 | **0.020** |
| Normalised stride time | 0.21 | 0.01 | 0.21 | 0.01 | 0.755 |
| Left stride length (% WH) | 198.35 | 15.70 | 206.27 | 18.08 | 0.198 |
| Right stride length (% WH) | 199.16 | 16.00 | 205.08 | 18.15 | 0.378 |
| Stride speed (m/s) | 2.19 | 0.19 | 2.45 | 0.29 | **0.012** |
| Normalised stride speed | 9.50 | 1.10 | 9.83 | 1.14 | 0.755 |
