# Supplementary material for: Biomechanical comparison of standing posture and during trot between German shepherd and Labrador retriever dogs
Source: PLoS One. 2020 Oct 2;15(10):e0239832. doi: 10.1371/journal.pone.0239832 (PMC7531786; doi:10.1371/journal.pone.0239832)
Supplement: S4 Table — P values less than 0.05 are in bold. (DOCX) [file pone.0239832.s004.docx]

**Table S4: The mean maximum and minimum values for flexion angles in the sagittal plane for the LRDs and GSDs during standing and trotting.** P values less than 0.05 are in bold.

|  | Standing | | | | | Trotting maximum | | | | | Trotting minimum | | | | |
| --- | --- | --- | --- | --- | --- | --- | --- | --- | --- | --- | --- | --- | --- | --- | --- |
| Kinematic parameter | LRD | | GSD | |  | LRD | | GSD | |  | LRD | | GSD | |  |
|  | Mean | SD | Mean | SD | P value | Mean | SD | Mean | SD | P value | Mean | SD | Mean | SD | P value |
| Neck flexion angle (°) | -15.39 | 5.22 | -18.72 | 8.68 | 0.319 | 5.24 | 8.21 | 5.90 | 9.24 | 1.000 | -5.06 | 8.40 | -6.14 | 10.64 | 0.887 |
| Mid-thoracic flexion angle (°) | -12.21 | 5.79 | -3.83 | 5.55 | **0.002** | 8.13 | 9.01 | 6.13 | 5.01 | 0.219 | -0.11 | 7.51 | -5.95 | 5.34 | **0.014** |
| Thoracolumbar flexion angle (°) | -1.35 | 6.06 | 0.89 | 3.88 | 0.291 | -3.96 | 3.02 | 5.54 | 5.21 | **<0.001** | -12.39 | 4.29 | -5.17 | 5.41 | **0.001** |
| Lumbosacral flexion angle (°) | 7.18 | 4.46 | 13.00 | 5.17 | **0.007** | 9.23 | 4.96 | 15.10 | 4.91 | **0.017** | 2.29 | 3.91 | 8.97 | 5.30 | **0.002** |
| Tail set flexion angle (°) | 45.32 | 8.63 | 32.46 | 4.96 | **<0.001** | 26.41 | 19.57 | 23.95 | 6.39 | 0.671 | 12.30 | 17.51 | 16.57 | 7.13 | 0.198 |
|  |  |  |  |  |  |  |  |  |  |  |  |  |  |  |  |
| Left hip flexion angle (°) | 31.54 | 14.21 | -9.21 | 11.19 | **<0.001** | 51.15 | 16.15 | 21.93 | 13.74 | **<0.001** | 18.53 | 16.20 | -19.99 | 14.88 | **<0.001** |
| Right hip flexion angle (°) | 27.61 | 10.22 | -10.02 | 10.12 | **<0.001** | 50.14 | 13.73 | 23.80 | 11.08 | **<0.001** | 14.77 | 11.98 | -20.44 | 11.99 | **<0.001** |
| Left stifle flexion angle (°) | 34.87 | 9.09 | 44.19 | 9.09 | **0.028** | 81.28 | 9.06 | 89.08 | 9.57 | **0.033** | 18.46 | 8.63 | 27.91 | 9.19 | **0.024** |
| Right stifle flexion angle (°) | 32.50 | 10.75 | 44.14 | 8.04 | **0.005** | 77.52 | 12.40 | 88.70 | 6.38 | **0.028** | 12.44 | 11.97 | 27.62 | 7.94 | **0.001** |
| Left hock flexion angle (°) | 29.58 | 8.74 | 58.35 | 11.36 | **<0.001** | 82.61 | 6.70 | 97.22 | 12.81 | **0.005** | 15.17 | 6.56 | 33.87 | 5.59 | **<0.001** |
| Right hock flexion angle (°) | 23.09 | 9.40 | 56.63 | 10.30 | **0.007** | 81.64 | 10.41 | 97.16 | 13.05 | **0.004** | 11.63 | 7.04 | 33.48 | 9.40 | **<0.001** |
|  |  |  |  |  |  |  |  |  |  |  |  |  |  |  |  |
| Left shoulder flexion angle (°) | 70.27 | 8.00 | 60.33 | 9.57 | **0.039** | 75.62 | 14.60 | 67.24 | 11.10 | 0.098 | 38.88 | 11.35 | 38.25 | 13.09 | 1.000 |
| Right shoulder flexion angle (°) | 76.09 | 8.18 | 59.44 | 9.05 | **<0.001** | 81.75 | 10.86 | 70.89 | 12.06 | 0.059 | 37.40 | 16.60 | 35.99 | 12.32 | 0.872 |
| Left elbow flexion angle (°) | 43.55 | 6.96 | 42.55 | 10.03 | 1.000 | 102.79 | 6.62 | 104.55 | 7.20 | 0.381 | 32.16 | 12.23 | 37.42 | 11.71 | 0.059 |
| Right elbow flexion angle (°) | 44.37 | 8.25 | 42.96 | 7.11 | 0.713 | 102.79 | 15.23 | 105.63 | 9.43 | 0.786 | 35.66 | 15.15 | 37.69 | 8.52 | 0.695 |
| Left carpal flexion angle (°) | 25.39 | 9.46 | 30.59 | 6.92 | 0.101 | 97.64 | 11.47 | 93.57 | 14.54 | 0.242 | -39.00 | 15.99 | -44.42 | 9.53 | 0.378 |
| Right carpal flexion angle (°) | 20.52 | 9.27 | 24.25 | 8.16 | 0.347 | 97.45 | 14.38 | 94.56 | 17.19 | 0.786 | -41.69 | 10.70 | -41.58 | 10.66 | 0.976 |
