# Supplementary material for: Biomechanical comparison of standing posture and during trot between German shepherd and Labrador retriever dogs
Source: PLoS One. 2020 Oct 2;15(10):e0239832. doi: 10.1371/journal.pone.0239832 (PMC7531786; doi:10.1371/journal.pone.0239832)
Supplement: S5 Table — Expressed as a percentage of the height at withers, which were measured using the positions of consecutive anatomical markers. P values less than 0.05 are highlighted in bold. For markers’ names see Table 1 in the manuscript. (DOCX) [file pone.0239832.s005.docx]

**Table S5: Bone and segment linear lengths of the LRDs and GSDs**. Expressed as a percentage of the height at withers, which were measured using the positions of consecutive anatomical markers. P values less than 0.05 are highlighted in bold. For markers’ names see Table 1 in the manuscript.

| Bone / segment length (markers’ location) | LRD | | GSD | |  |
| --- | --- | --- | --- | --- | --- |
|  | Mean (%) | SD (%) | Mean (%) | SD (%) | P value |
| Cranial thorax (With.- T8) | 19.41 | 3.72 | 19.39 | 5.49 | 1.000 |
| Caudal thorax (T8 - L1) | 23.76 | 3.82 | 20.96 | 5.35 | 0.089 |
| Lumbar vertebrae (L1 - L5) | 17.51 | 3.39 | 20.35 | 3.03 | **0.028** |
| Thoracolumbar (With. – S1) | 77.79 | 5.00 | 86.11 | 7.64 | **0.008** |
|  |  |  |  |  |  |
| Pelvis (S1 - GT) | 27.73 | 3.93 | 27.03 | 2.63 | 0.977 |
| Left femur | 34.85 | 4.20 | 31.84 | 2.58 | 0.089 |
| Right femur | 36.08 | 4.28 | 32.92 | 2.64 | **0.020** |
| Left tibia | 34.52 | 3.03 | 30.97 | 2.46 | **0.005** |
| Right tibia | 32.60 | 2.54 | 30.48 | 2.07 | **0.021** |
| Left metatarsal bones | 18.39 | 1.03 | 19.06 | 1.74 | 0.410 |
| Right metatarsal bones | 19.19 | 1.08 | 18.24 | 2.53 | **0.024** |
|  |  |  |  |  |  |
| Left humerus | 29.95 | 1.99 | 25.55 | 2.92 | **0.001** |
| Right humerus | 31.09 | 2.80 | 26.53 | 3.10 | **0.002** |
| Left radius | 36.58 | 2.41 | 34.53 | 2.04 | **0.010** |
| Right radius | 37.32 | 2.32 | 34.26 | 1.40 | **0.002** |
| Left metacarpal bones | 11.18 | 2.09 | 10.74 | 1.95 | 0.551 |
| Right metacarpal bones | 11.66 | 1.73 | 10.86 | 1.87 | 0.316 |
